# Dimensionality of the Pittsburgh Sleep Quality Index: a systematic review

**DOI:** 10.1186/s12955-018-0915-x

**Published:** 2018-05-09

**Authors:** Md Dilshad Manzar, Ahmed S. BaHammam, Unaise Abdul Hameed, David Warren Spence, Seithikurippu R. Pandi-Perumal, Adam Moscovitch, David L. Streiner

**Affiliations:** 1 Department of Nursing, College of Applied Medical Sciences, Majmaah University, Al Majmaah, Kingdom of Saudi Arabia; 2 Department of Biomedical Sciences, College of Medicine and Health Sciences, Mizan-Tepi University (Mizan Campus), Mizan-Aman, Ethiopia; 3 The University Sleep Disorders Center, Department of Medicine, College of Medicine, King Saud University, Riyadh, Saudi Arabia; 4 The National Plan for Science and Technology, King Saud University, Riyadh, Saudi Arabia; 5 Department of Physiotherapy, Fatima College of Health Sciences, Al Mafraq, Abu Dhabi City, United Arab Emirates; 6 Toronto, Canada; 7 Sleep and Fatigue Institute, University of Calgary, Calgary, AB Canada; 8 Department of Psychiatry and Behavioral Neurosciences, McMaster University, Hamilton, ON Canada; 9 Department of Psychiatry, University of Toronto, Toronto, ON Canada

**Keywords:** Confirmatory factor analysis, Dimensionality, Exploratory factor analysis, Model fit, Systematic review, Sleep

## Abstract

**Background:**

The Pittsburgh Sleep Quality Index (PSQI) dimensionality is much debated, with the greatest number of reported factor structures. Therefore, this review appraised the methodologies of studies investigating the factor structure of the PSQI.

**Material and methods:**

MEDLINE, PsycInfo, AJOL, BASE, Cochrane Library, Directory of Open Access Journals (Lund University), CINAHL, and Embase were searched systematically to include articles published till 23rd March, 2018. The articles with the objective of factor analysis of the PSQI (20 articles) or with a major section on the same subject (25 articles) were included. There was no limitation about participant characteristics. Descriptive analysis of articles for measures of the suitability of the data for factor analysis, details of the exploratory factor analysis (EFA) and details of the confirmatory factor analysis (CFA) was performed.

**Results:**

The analysis used by the majority did not employ the simplest scheme for interpreting the observed data: the parsimony principle. Other shortcomings included under- or non-reporting of sample adequacy measures (11 out of 45 articles), non-use of EFA (20 out of 45 articles), use of EFA without relevant details, non-use of CFA (11 out of 45 articles), and use of CFA without relevant details. Overall, 31 out of 45 articles did not use either EFA or CFA.

**Conclusion:**

We conclude that the various PSQI factor structures for standard sleep assessment in research and clinical settings may need further validation.

**Trial registration:**

Not applicable because this was a review of existing literature.

## Background

Population-based epidemiological studies have confirmed that sleep disorders occur frequently in almost every country [1–3]. Complaints of disturbed or poor quality sleep are also exceedingly common among patients presenting to all specialties of medicine [4–6]. The most common sleep disorders are insomnia, circadian rhythm sleep disorders, obstructive sleep apnea, sleep-disordered breathing, hypersomnia, daytime sleepiness, parasomnias, and restless legs syndrome [4–7]. Untreated sleep disorders may lead to potentially life-threatening symptoms. It is now recognized that far from being only a consequence of medical illnesses, sleep disorders are often primary drivers of other illnesses. Sleep disturbance is linked to neurocognitive dysfunctions, including attention deficits, impaired cognitive performance, depression, anxiety, stress, and poor impulse control. These disturbances are in turn linked to sympathetic activity changes and an increased risk of cardiovascular and cerebrovascular diseases [4, 5, 8]. These impairments have wider consequences in patients’ lives. Poor sleep severely impairs daytime performance, both socially and at work, and increases the risk of occupational and automobile accidents, poor quality of life, and poor overall health [4, 5, 9–11].

### Role of subjective measurement

The ever-increasing list of problems known to be caused by sleep dysfunction has led to recognition that poor sleep has a complex relationship with overall health. It is now appreciated that disturbed sleep interacts bi-directionally with numerous neurological, physiological, psychological, and behavioral factors [4, 12–14]. The central role of sleep in overall health has thus underscored the need for both reliable, validated subjective tools and objective polysomnographic (PSG) assessment in modern medical practice. While these represent very different diagnostic approaches, they are nevertheless complementary in as much as subjective tools account for psychological and behavioral manifestations not assessed by PSG. Self-rating questionnaires such as the Pittsburgh Sleep Quality Index (PSQI) have an important role in sleep health assessment in both clinical and research settings [4, 15, 16]. These questionnaires have the advantages of cost effectiveness, high patient compliance, and ease of administration. Perhaps more importantly, since such questionnaires are self-explanatory and do not require supervision, they reduce demand on medical specialists’ time [5]. Given the important diagnostic role of rating scale questionnaires, it is essential that their reliability and validity be established beyond doubt. A key element of this quality assurance is psychometric confirmation of the questionnaires’ dimensionality, i.e., whether the questionnaire’s items are all correlated and representative of factors affecting sleep quality [4, 15]. This review critically appraises the evidence for dimensionality of one of the most widely used self-rating instruments of sleep quality, the PSQI [4, 15, 17].

### Pittsburgh Sleep Quality Index

The PSQI is the most widely used sleep health assessment tool in both clinical and non-clinical populations. The original 1989 article describing the Index has, since 26-06-2015, had 1545, 7863, 4962, and 4554 citations on PMC, Google Scholar, ResearchGate, and Web of Science, respectively. It is also possibly the most widely translated sleep questionnaire. The PSQI consists of 24 questions or items to be rated (0–3 for 20 items while 4 items are open-ended), 19 of which are self-reported and 5 of which require secondary feedback from a room or bed partner. Only the self-reported items (15 rated as 0–3 while 4 open-ended) are used for quantitative evaluation of sleep quality as perceived by the patient. The open-ended items are also finally scored as structured categorical values (rated at 0–3) as per the range of values reported for them by the patient. These 19 self-reported items are used to generate categorical scores representing the PSQI’s 7 components. The individual component scores each assess a specific feature of sleep. Finally, the scores for each component are summed to get a total score, also termed the global score (range: 0 to 21). This score provides an efficient summary of the respondent’s sleep experience and quality for the past month [12].

### Validation and reliability measures of the Pittsburgh Sleep Quality Index

The PSQI is possibly the most rigorously validated tool used in sleep diagnostics [4, 5, 15–17]. Of the many psychometric studies carried out on the PSQI, 75% have reported an internal consistency in the ideal range for within- and between-group comparisons but not for comparisons made between questionnaires for individual patients [4]. Mollayeva et al. [4] performed a meta-analysis and found strong evidence for the PSQI’s reliability and validity. Further, the meta-analysis revealed a moderately positive evidence for the questionnaire’s structural validity across a variety of samples. The PSQI was found to have known-group validity, and, while some studies showed methodological weaknesses in this regard, its convergent and divergent validity were generally confirmed.

### Factor analysis

A tool’s dimensionality is evaluated by factor analysis. Factor analysis attempts to discover patterns in a set of variables based on shared variance [18]. A key goal of this analysis is identifying the simplest and most parsimonious means for interpreting and representing observed data [19]. More specifically, the procedure seeks to use measured variables to infer the smallest number of unobserved or latent variables that can still account for the observable variables [20]. The mathematical operations are broadly categorized into 2 sub-groups: exploratory factor analysis (EFA) and confirmatory factor analysis (CFA). EFA aims to find the smallest number of common latent factors that can account for the correlations [21]. CFA is then employed to test the relationship between the observed variables and their underlying latent factors [15]. Factor analysis is useful for studies involving many variables that can be reduced to a smaller set, such as questionnaire items or a battery of tests. The goal of this process is identifying the concepts underlying a phenomenon and thus facilitating interpretations.

### Dimensionality of the PSQI

The PSQI’s dimensionality is much debated, with many studies supporting multiple factors and some supporting unidimensionality [4, 15–17]. Among sleep diagnostic tools, the PSQI has the greatest number of reported factor structures [15]. The intensity of discussion around the topic of the dimensionality of the PSQI is reflected in the publication of 45 articles on the subject since 2006 [15]. As the PSQI components are structured categorical variables scored 0–3, therefore the factor analysis should ideally begin with a polychoric correlation matrix. However, most of the programs do use a Pearson correlation matrix. It may be one of the reasons for the discrepancy among studies. Some evidence suggests that some studies may have over-factored the PSQI [15]. Several reviews have concluded that many previous efforts to investigate the PSQI’s factor structure have suffered from non-parsimonious methodologies [4, 15, 17, 22–46]. Given a choice between close fit and parsimony (i.e., model with fewer latent factors), the latter may be preferred [47]. Manzar et al. [15] used an innovative strategy of performing comparative CFA of all the documented PSQI models on a discrete sample to disprove the questionnaire’ soft-reported multidimensionality and heterogeneity. However, the study had the important limitation of being unable to address inter-software, inter-sample, and inter-model differences [15]. Mollayeva et al. [4] mentioned procedural discrepancies in the studies investigating the PSQI factor structures without providing further details. Approximately 30 distinct PSQI models have been proposed in the literature. Of these models, 7 were 1-factor, 17 were 2-factor, 4 were 3-factor, 1 was 4-factor and 2 were second order models [15–17, 22–46, 48–53]. The current state of the literature, with its broad range of suggested factor structure models, represents an impediment to an efficient consideration of the PSQI’s use. There evidently exists a need for a thorough appraisal of the procedural details and application of standard practices in the previous methodological studies of the PSQI. Such an investigation is indispensable for streamlining the debate about the PSQI’s heterogeneity.

### Practical implication of the heterogeneity of the Pittsburgh Sleep Quality Index

One consequence of the PSQI’s presumed heterogeneity is the possible attenuation of its practical application in clinical diagnostics [15]. A questionnaire’s dimensionality directly affects the reporting of its intended measures. Currently, however, very few efforts have been undertaken to validate the PSQI’s disparate models in either research or clinical settings. This is possibly related to the choice of the appropriate PSQI model for a particular sample. Previous attempts by Hancock and Larner [54] and Yurcheshen et al. [55] to test the disparate PSQI models did not adequately address the reason(s) for the specific model’s selection. In fact, both studies used a 3-factor PSQI model initially reported to be valid in a different population [22, 54–56]. Such reports using unrelated PSQI models will complicate inter-study comparisons for the PSQI-based measures. The goal of the present systematic review is to help develop strategies for managing the methodological discrepancies in the PSQI factor analysis and reporting of the PSQI-based sleep assessment. An additional goal is to provide possible guidelines for factor analyses of questionnaires in general and sleep inventories in particular.

## Material and methods

### Literature search scheme

All articles available online on 23-03-2018 were included. The comprehensive search strategy was planned in consultation with epidemiological experts, information technologists, and sleep scientists. We searched 8 electronic databases: CINAHL, Bielefeld University (BASE: Bielefeld Academic Search Engine), Cochrane Library, Directory of Open Access Journals (Lund university), Embase, Medline, PsycInfo, and African Journals On Line (AJOL).

**Fig. 1:**
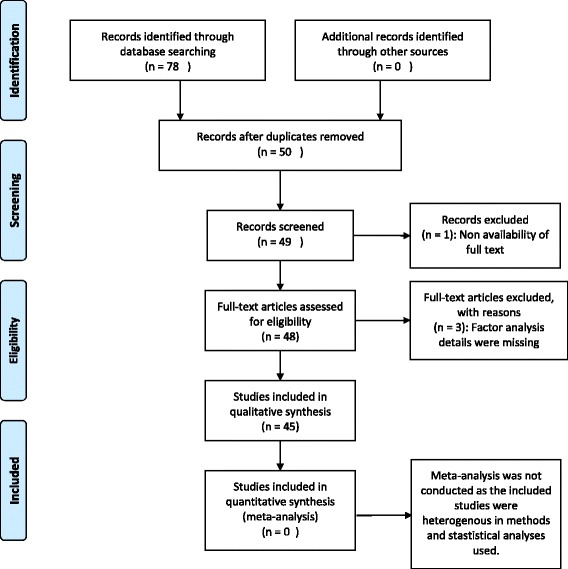
Schematic of the article selection process

To minimize inclusion of irrelevant articles, we searched for a combination of 2 keywords (Pittsburgh Sleep Quality Index/PSQI with dimensionality/dimension/factor model/factor analysis/factor structure/domain/Exploratory factor analysis/EFA/Confirmatory factor analysis/CFA). Seventy-eight articles were initially identified (Fig. 1). Thirty four articles; 30 duplicates, 3 with reasons (Factor analysis details were missing) and 1 for unavailability of full-length article were removed.

### Selection criteria

Forty five full-length peer-reviewed articles were used. Forty three articles were in English, and 1 each was in Spanish and Chinese. We e-mailed the lead author of the Spanish article an English translation of the section covering factor analysis. It was included after gaining the author’s approval. The lead author of the Chinese article provided translation of the factor analysis section, therefore it was also included. The articles’ reference lists were thoroughly reviewed for other relevant publications. There were no restrictions on the type or age range of the population covered. We only included articles that had a primary objective of exploring and/or confirming dimensionality (20 studies) and articles that reported multiple indices of psychometric properties with a substantial section devoted to factor analysis (25 studies) (Fig. 1).

### Data extraction

The measures used to present the factor analysis findings were grouped in three broad categories; measures of the suitability of the data for factor analysis (Table 1), summary of the exploratory factor analysis conditions (Table 2) and summary of the confirmatory factor analysis conditions (Table 3). Descriptive analysis of articles for measures of these three categories was performed. Meta-analysis was not conducted as the included studies were heterogeneous in methods and statistical analyses used.

**Table 1:** Sample description, sample size, and measures of the suitability of the data for factor analysis in the studies reporting factor structure of the Pittsburgh Sleep Quality Index

Author and year of publication	Sample description, age	Sample size	KMO & Bartlett’s Test of Sphericity	Determinant Score & Anti-image/Diagonal element of anti-correlation matrix	Inter-component co-relations
Aloba et al. 2007 [31]	Nigerian College students	520	–	–	–
Anandakumar et al. 2016 [67]	outpatients at a hospital in Srilanka, 50.02 ± 13.5	205	0.83 & < 0.001	–	0.42–0.81
Babson et al. 2012 [30]	Military veterans with PTSD	226	–	–	− 0.02–0.53
Becker & Jesus 2017 [53]	Community dwelling Portugese adults, 70.05 ± 7.15	204	0.731 & < 0.001	–	0.12–0.52
Benhayon et al. 2013 [61]	Pediatric patients with Crohn disease (14.4 ± 2.3) and healthy controls (14.8 ± 2.0)	CD, *n* = 96 Healthy controls. *N* = 19	0.7 & < 0.001	–	–
Burkhalter et al. 2010 [29]	American renal transplant recipients	135	–	–	0.14–0.73
Buysse et al. 2008 [28]	Senior African-American & Caucasian adults	187	–	–	–
Casement et al. 2012 [35]	Women with PTSD	319	–	–	–
Chong & Cheung 2012 [34]	Cantonese Chinese, age > 45 years	794	–	–	0.07–0.76
Qiu et al. 2016 [58]	Pregnant women, 33.4 ± 4.2	1488	0.72 & < 0.001	–	–
Cole et al. 2006 [22]	American older adults	207, 210	–	–	0.04–0.60, 0.11–0.66
De la Vega et al. 2015 [59]	Adolescents and young adults, 17.12 ± 3.05	216	.77 & < 0.001	–	–
DeGutis et al. 2016 [62]	Trauma exposed veterans, 31.51 ± 8.16	283	–	–	–
Dudysova et al. 2017 [66]	Outpatients of the sleep laboratory at Prague psychiatric center, 44.5 ± 14.24	105	–	–	0.05–0.73
Gelaye et al. 2014 [44]	Chilean, Ethiopian, Peruvian and Thai college students	(*N* = 830), (*N* = 2230), (*N* = 2581), (*N* = 2840)	0.724 to 0.801 & < 0.001	–	–
Hita-Contreras et al. 2014 [43]	Spanish fibromyalgia patients	138	0.784 & < 0.001	–	–
Ho et al. 2014 [42]	Chinese breast cancer patients	197	–	–	0.15–0.78
Jiménez-Genchi et al. 2008 [27]	Spanish healthy controls and psychiatric patients	135	–	–	0.06–0.77
Jomeen& Martin 2007 [26]	Pregnant women with depression, 28.86 ± 55.19	180	–	–	–
Koh et al. 2015 [41]	Multi-ethnic Asians in Singapore	489, 1976	–	–	0.02–0.48, 0.05–0.36
Kotronoulas et al. 2011 [25]	Cancer patients on chemotherapy	209	.79 & < 0.001	–	.16–.70
Lequerica et al. 2014 [40]	Traumatic brain injury patients	243	–	–	–
Magee et al. 2008 [24]	Australian adults	364	–	–	< 0.43
Manzar et al. 2016a [17]	Indian university students	209, 209	0.754 & < 0.001	> 0.00001 & all values > 0.5	–
Manzar et al. 2016b [15]	Indian university students	418	0.758 & < 0.001	> 0.00001 & all values > 0.5	–
Mariman et al. 2012 [33]	Belgians with CFS	413	–	–	0.02–0.72
Nazifi et al. 2014 [39]	Iranian health professionals	415	0.58 & < 0.05	–	–
Nicassio et al. 2014 [38]	American rheumatoid arthritis patients	107	–	–	–
Otte et al. 2013 [32]	Breast cancer survivors	1172	–	–	0.18–0.51
Otte et al. 2015 [37]	Perimenopausal and postmenopausal women with hot flashes	849	–	–	0.01–0.46
Rener-Sitar et al. 2014 [46]	TMD, 37.1 ± 13.1	TMD with pain (496) & TMD without pain (113)	–	–	− 0.18 to 0.74
Salahuddin et al. 2017 [16]	Commmunity dwelling Ethiopian Adults, 25.5 ± 6.0	311	0.51, 0.52 & < 0.001, < 0.001	0.08, 0.09 & 0.39–0.67 0.48–0.64	–
Skouteris et al. 2009 [23]	Pregnant women with depression, 31.67 ± 4.55	252	–	–	–
Tomfohr et al. 2013 [36]	Community-dwelling English speaking Spanish, English and Non-hispanic white	792, 654, 1698	–	–	0.05–0.48, 0.18–0.59, 0.07–0.52
Yunus et al. 2016 [48]	Community dwelling older Malaysians	Phase 1, *n* = 183 Phase 2, *n* = 2118	–	–	–
Zheng et al. 2016 [50]	Chinese medical students, 20.2 ± 1.3	603	–	–	0.45–0.57
Zhong et al. 2015 [45]	Pregnant Peruvian women	642	0.65 & < 0.001	–	0.10 to 0.40
João et al. 2017 [57]	Portuguese community-dwelling adults, 35.93 ± 11.01	347	0.59 & < 0.001	–	0.00–0.54
Chen et al. 2017 [63]	Taiwanese insomniacs, 43.15	114	–	–	–
Khosravifar et al. 2015 [51]	Depressed and healthy Iranians, 32.3 ± 7.1	193	0.598 & < 0.001	–	–
Fontes et al. 2017 [49]	Portuguese breast cancer patients, 57.9 ± 10.8	474	–	–	–
Guo et al. 2016 [60]	Chinese undergraduate students,20.86 ± 1.33	631	–	–	–
Morris et al. 2017 [65]	Diabetic Americans male and females, 55.3 ± 11.1, 58.5 ± 10.0	198	0.60 & < 0.001	–	< 0.80
Passos et al. 2016 [52]	Brazilian adolescents, 10–19	309	0.59 & < 0.001	–	–
Zhu et al. 2018 [64]	Chinese adults with type 2 diabetes, 55.18 ± 12.65	240	0.82 & < 0.01	–	0.05–0.65

**Table 2:** Summary of the exploratory factor analysis conditions used and reported by the studies investigating the factor structure of the Pittsburgh Sleep Quality Index

Author and year of publication	Extraction test	Rotation	Scree plot reported (Y/N), Total variance reported (Y/N), Eigen value rule (Y/N), Robust measure of factor retention (Y/N)	Number of factors	Pattern matrix reported (Y/N)	Communality reported (Y/N)
Aloba et al. 2007 [31]	Principal component analysis	Not reported	N, N, N, N	3	Y	–
Anandakumar et al. 2016 [67]	principal components analysis	Not reported	N, Y, N, N	1	Y	–
Babson et al. 2012 [30]	Not reported	Standardized geomin rotation	N, N, N, N	2	Y	–
Becker & Jesus 2017 [53]	maximum likelihood estimation	direct oblimin rotation	N, Y (40.56%), N, N	2	Y	N
Benhayon et al. 2013 [61]	principal axis factoring method	direct oblimin rotation	Y, N, Y, N	2	Y	N
Burkhalter et al. 2010 [29]	NO EFA	–	–	–	–	–
Buysse et al. 2008 [28]	Principal components analysis	Varimax rotation	N, N, Y, N	2	Y	–
Casement et al. 2012 [35]	NO EFA	–	–	–	–	–
Chen et al. 2017 [63]	No EFA	–	–	–	–	–
Chong & Cheung 2012 [34]	NO EFA	–	–	–	–	–
Cole et al. 2006 [22]	Principal components analysis & maximum likelihood estimation	Direct oblimin rotation	N, Y (57.3%), N, N	2	Y	N
De la Vega et al. 2015 [59]	No EFA	–	–	–	–	Y, 0.42–0.66
DeGutis et al. 2016 [62]	No EFA	–	N, N, N, N	–	–	N
Dudysova et al. 2017 [66]	No EFA	–	–	–	–	–
Fontes et al. 2017 [49]	Principal component analysis	Varimax with Kaiser Normalization rotation	N, Y (38, 57%), Y, N	1, 2	Y	–
Gelaye et al. 2014 [44]	Principal component analysis	Orthogonal rotation	Y, Y, Y, N	2 & 3	Y	N
Guo et al. 2016 [60]	No EFA	–	–	–	–	–
Hita-Contreras et al. 2014 [43]	Principal component factor analysis	Varimax rotation	N, Y (54.96%), Y, N	2	Y	Y, 0.21 to 0.71
Ho et al. 2014 [42]	NO EFA	–	–	–	–	–
Jiménez-Genchi et al. 2008 [27]	Principal components analysis	Not reported	N, Y (63.2%), Y, N	2	Y	N
João et al. 2017 [57]	Principal components analysis	Not reported	N, Y (26.47%), N, N	1	Y	–
Jomeen& Martin 2007 [26]	NO EFA	–	–	–	–	–
Khosravifar et al. 2015 [51]	principal component	Oblimin rotation	N, Y (58.3%), Y, N	2	Y	–
Koh et al. 2015 [41]	Principal component analysis & maximum likelihood estimation	Varimax rotation	N, N, N, N	3	–	–
Kotronoulas et al. 2011 [25]	Principal component analysis	Direct oblimin rotation	N, Y (59.2%), Y, N	2	Y	Y, 0.38 to 0.75
Lequerica et al. 2014 [40]	Maximum likelihood estimation	Promax rotation	N, Y (62.4%), Y, N	2	Y	N
Magee et al. 2008 [24]	Principal component analysis with maximum likelihood estimate extraction	Direct oblimin rotation	N, Y, N, N	2	Y	N
Manzar et al. 2016a [17]	Principal component analysis & maximum likelihood estimation	Direct oblimin rotation	Y, Y (51.27%), Y, Parallel analysis	2& 1	Y	Y, 0.39–0.64
Manzar et al. 2016b [15]	NO EFA	–	–	–	–	–
Mariman et al. 2012 [33]	NO EFA	–	–	–	–	–
Morris et al. 2017 [65]	Principal components analysis	varimax & Promax rotation	Y, Y (68.08%, 74.11), Y, Y, Parallel analysis	3	Y	–
Nazifi et al. 2014 [39]	Principal components analysis	Varimax rotation	N, Y (63.485%), N, N	3	N	N
Nicassio et al. 2014 [38]	NO EFA	–	–	–	–	–
Otte et al. 2013 [32]	NO EFA	–	–	–	–	–
Otte et al. 2015 [37]	NO EFA	–	–	–	–	–
Passos et al. 2016 [52]	Not reported	varimax orthogonal	N, Y (66.57, 52.07, 60.41%), N, N	3, 2, 2	Y	–
Qiu et al. 2016 [58]	principal component analysis	oblique promax rotation	Y, Y (52.8%), Y, N	2	N	N
Rener-Sitar et al. 2014 [46]	Principal factors method	Orthogonal varimax or oblique promax	Y, Y, Y, N	1	Y	–
Salahuddin et al. 2017 [16]	maximum likelihood estimation	direct oblimin	Y, Y, Y, Y	1, 2, 3	Y	–
Skouteris et al. 2009 [23]	NO EFA	–	–	–	–	–
Tomfohr et al. 2013 [36]	NO EFA	–	–	–	–	–
Yunus et al. 2016 [48]	No EFA	–	–	–	–	–
Zheng et al. 2016 [50]	No EFA	–	–	–	–	–
Zhong et al. 2015 [45]	principal component analysis	promax rotation	N, Y (60.10%), Y, N	3	Y	N
Zhu et al. 2018 [64]	No EFA	–	–	–	–	–

**Table 3:** Summary of the confirmatory factor analysis conditions used and reported by the studies investigating the factor structure of the Pittsburgh Sleep Quality Index

Author and year of publication	Software	Extraction method	Types of Modification index used	Correlation between factors	Standardized Factor loadings	Factors in final model; same/different from EFA	Number of models used in comparative CFA	Reason for the selection of models in comparative CFA	Model fit indices
Aloba et al. 2007 [31]	NO CFA	–	–	–	–	–	–	–	–
Babson et al. 2012 [30]	NO CFA	–	–	–	–	–	–	–	–
Burkhalter et al. 2010 [29]	Mplus version 5.21	Not reported	Path diagram change	0.532, 0.773, 0.801	F1 DURAT = 0.85, HSE = 0.98, SLPQUAL = − 0.51 F2 SLPQUAL = 1.09, LATEN = 0.68, MEDS = 0.92 F3 DISTB = 0.93, DAYDYS = 0.56	3, No EFA	3; 1F-1 3F-2	Not explained	Non-significant *p* value of χ^2^; RMSEA< 0.08–0.05; CFI > 0.95; WRMR < 0.90.
Buysse et al. 2008 [28]	NO CFA	–	–	–	–	–	–	–	–
Casement et al. 2012 [35]	Mplus version 5.1	Mean and variance-adjusted weighted least squares (WLSMV) estimator	Not reported	0.46, 0.77, 0.81	F1 DURAT = 0.87, HSE = 0.75 F2 SLPQUAL = 0.75, LATEN = 0.56, MEDS = 0.45 F3 DISTB = 0.74, DAYDYS = 0.43	3, No EFA	4; 1F-1 2F-2 3F-1	Not explained, some of the documented models not used, no reasons given for selection and/or inclusion	χ^2^/ df < 3, RMSEA < 0.06, WRMR < 0.90, CFI ≥ 0.95, and TLI ≥ 0.96
Chong & Cheung 2012 [34]	Mplus version 5	Not reported	Not reported	0.522, 0.567, 0.641	F1 DURAT = 0.73/0.85/0.95, HSE = 0.76/0.84/0.78 F2 SLPQUAL = 0.81/0.59/0.63, LATEN = 0.64/0.64/0.70, DISTB = 0.59/0.40/0.47, DAYDYS = 0.44/0.21/49, MEDS = 0.33/0.35/0.17	2, No EFA	9; 1F-1 2F-6 3F-2	Partially explained, some of the documented models not used, no reasons given for their omission	SRMR< 0.05; RMSEA < 0.07; CFI > 0.95
Cole et al. 2006 [22]	Not reported	Maximum likelihood extraction on the covariance matrix, & multivariate non-normality smoothed by bootstrapping	Lagrange Modification index with change in path diagram	0.42, 0.82, 0.75	F1 DURAT = 0.76, HSE = 0.91 F2 SLPQUAL = 0.89, LATEN = 0.67, MEDS = 0.43 F3 DISTB = 0.67, DAYDYS = 0.52	2, 3	2; 2F-1 3F-1	Comparison between originally proposed 1F model & outcome of EFA Fit indices for 1F model not reported	RMSEA≤0.06; CFI ≥ 0.90; GFI ≥ 0.90; AGFI≥0.90; LOWER χ^2^, BIC (difference of at least 10 between two models)
Gelaye et al. 2014 [44]	Stata version 12.0 software	Maximum likelihood estimation	Not reported	0.46, 0.26, 0.36, (0.53, 0.40, 0.10)	F1 DURAT = 0.79/0.73/1.0/0.6, HSE = 0.43/0.78/0.21/0.57 F2 SLPQUAL = 0.81/0.58/0.61/0.67, LATEN = 0.47/0.35/0.34/0.53, DISTB = 0.47/0.51/0.54/0.38, DAYDYS = 0.49/0.51/0.5/0.39, MEDS = 0.25/0.25/0.14/0.28	2, 2, 2, 3, same	Not performed	Not explained	SRMR ≤0.08; RMSEA ≤0.06; CFI ≥0.95
Hita-Contreras et al. 2014 [43]	NO CFA	–	–	–	–	–	–	–	–
Ho et al. 2014 [42]	Mplus version 7.11	Robust maximum likelihood estimator	Error-term correlation	Not applicable	F1 DURAT = 0.59, HSE = 0.60, SLPQUAL = 0.84, LATEN = 0.61, DISTB = 0.61, DAYDYS = 0.56, MEDS = 0.36	1, same	4; 1F-2 2F-1 3F-1	Partially explained, some of the documented models not used, no reasons given for their omission	Insignificant χ^2^-test; CFI & TLI ≥0.95; RMSEA≤0.06; SRMR≤0.08; Lower BIC
Jiménez-Genchi et al. 2008 [27]	NO CFA	–	–	–	–	–	–	–	–
Jomeen & Martin 2007 [26]	Mplus version 3	Weighted least-square with mean and variance correction estimator (WLSMV)	Not reported	Not reported	not reported	2, No EFA	7; 1F-1, 2F-6	Not clear	CFI & TLI > 0.90, RMSEA< 0.08–0.05, WRMR< 0.90 & Insignificant χ^2^
Koh et al. 2015 [41]	FactoMineR in R	Not reported	Not reported	(0.27, 0.64, 0.89); (0.39, 0.72, 0.90) in 2 sample groups	F1 DURAT = 0.68/0.60, HSE = 0.72/0.67 F2 SLPQUAL = 0.72/0.63, LATEN = 0.63/0.60 F3 DISTB = 0.37/0.52, DAYDYS = 0.51/0.42, MEDS = 0.40/0.26	3/3, 3/3, same	4; 1F-1 2F-1 3F-2	Not explained	GFI > 0.90; AGFI> 0.90; CFI ≥ 0.95 RMSEA < 0.08–0.05; LOWER χ^2^, BIC (difference of at least 10 between two models), CAIC
Kotronoulas et al. 2011 [25]	NO CFA	–	–	–	–	–	–	–	–
Lequerica et al. 2014 [40]	SPSS Statistics 21 with AMOS	Not reported	Not reported	0.87, 0.85	F1 DURAT = 0.68, HSE = 0.51, LATEN = 0.68 F2 DISTB = 0.73, DAYDYS = 0.66, MEDS = 0.25	2, same	5; 1F-1 2F-3 3F-1	Not explained, some of the documented models not used, no reasons given for selection and/or inclusion	Non-significant *p* value of χ^2^; CFI ≥ 0.95; NNFI≥0.95 RMSEA < 0.06
Magee et al. 2008 [24]	SPSS version 15 with AMOS version-7	Not reported	Not reported	0.73	F1 DURAT = 0.68, HSE = 0.62 F2 SLPQUAL = 0.76, LATEN = 0.61, DISTB = 0.46, DAYDYS = 0.52, MEDS = 0.23	2, different	6; 1F-2 2F-2 3F-2	Partially explained, some of the documented models not used, no reasons given for their omission	χ^2^-test lower, non-significant values; RMSEA ≤0.05; CFI, GFI, & AGFI > 0.90
Manzar et al. 2016a [17]	SPSS 16.0 with amos	Maximum likelihood extraction with bootstrapping to smooth non-normality	Not reported	Not applicable	F1 DURAT = 0.74, HSE = 0.32, SLPQUAL = 0.74, LATEN = 0.63, DISTB = 0.43, DAYDYS = 0.41, MEDS = 0.40	1, 2 different	2; 1F-1 2F-1	Comparison between outcome(s) of EFA	Non-significant Bollen–Stine bootstrap χ2 *p* values, Non-significant *p* value of χ^2^; χ2/df < 2; RMR ≤ 0.05; CFI ≥ 0.95; RMSEA < 0.05; GFI & AGFI> 0.9; AIC = lesser value indicated a better fit
Manzar et al. 2016b [15]	SPSS 16.0 with amos	Maximum likelihood extraction	Co-variance, Variance and regression weights	Not applicable	F1 DURAT = 0.363, HSE = 0.374, SLPQUAL = 0.705, LATEN = 0.633, DISTB = 0.501, DAYDYS = 0.406, MEDS = 0.30	1, No EFA	17; 1F-3 2F-8 3F-6	Most of models of the PSQI reported till 15–02-2015	Non-significant *p* value of χ^2^; χ2/df < 2; RMR ≤ 0.05; CFI ≥ 0.95; RMSEA < 0.05; GFI & AGFI> 0.9; AIC = lesser value indicated a better fit
Mariman et al. 2012 [33]	SPSS (PASW 17.0) with AMOS module (5.0)	Maximum Likelihood Algorithm	Not reported	0.64, 0.53, 1.00	F1 DURAT = 0.9, HSE = 0.78 F2 SLPQUAL = 0.85, LATEN = 0.57, MEDS = 0.18 F3 DISTB = 0.79, DAYDYS = 0.29 But, 3 latent factors shown to load on 1 factor	Second order model, No EFA	3; 1F-1 2F-1 3F-1 Results for the 2F model not shown	Not explained, some of the documented models not used, no reasons given for selection and/or inclusion	Non-significant *p* value of χ^2^ (d.f.); GFI > 0.90; AGFI> 0.85; CFI > 0.90; RMSEA< 0.08–0.05; Lower CAIC
Nazifi et al. 2014 [39]	NO CFA	–	–	–	–	–	–	–	–
Nicassio et al. 2014 [38]	EQS 6.1	Maximum likelihood (ML) method	Not reported	0.65	F1 DURAT = 0.85, HSE = 0.64 F2 SLPQUAL = 0.89, LATEN = 0.48, DISTB = 0.57, DAYDYS = 0.56	2, No EFA	3; 1F-1 2F-1 3F-1	Not explained, some of the documented models not used, no reasons given for selection and/or inclusion	S-Bχ^2^; an S-Bχ^2^/df < 2.0; robust CFI ≥ 0.95; RMSEA≤0.05; Lower & negative AIC
Otte et al. 2013 [32]	LISREL 8.8	Weighted least squares	Error term correlation	0.37, 0.71 in 2 sample groups	F1 DURAT = 0.64, HSE = 0.97 F2 SLPQUAL = 0.86, LATEN = 0.82/0.66, DISTB = 0.66, DAYDYS = 0.5, MEDS = 0.46	2, No EFA	4; 1F-1 2F-1 3F-2 Two 3F models differed with respect to use/non-use of error terms only	Not explained	Non-significant *p* value of χ^2^; SRMR ≤0.08; RMSEA< 0.06; CFI ≥ 0.95
Otte et al. 2015 [37]	LISREL version 8.8	Weighted least-squares, none of the indicators showed excessive skew or kurtosis	Not reported	0.40, 0.73, 0.68	F1 DURAT = 0.92, HSE = 0.68 F2 SLPQUAL = 0.82, LATEN = 0.57, MEDS = 0.15 F3 DISTB = 0.61, DAYDYS = 0.61	3, No EFA	7; 1F-1 2F-2 3F-3 4F-1	Not explained	Non-significant *p* value of χ^2^; RMSEA< 0.06; CFI ≥ 0.95;
Rener-Sitar et al. 2014 [46]	STATA version 12	Diagonally weighted least squares (DWLS) and a “robust” method using the Huber-White sandwich estimator	Not reported	Not applicable	not reported	1; same in both	Not applicable	Not applicable	SRMR: ≤0.08; RMSEA: ≤0.06; and CFI, TLI: ≥0.95
Skouteris et al. 2009 [23]	Structural equation modeling (SEM)	Not reported	Path diagram change	0.44, 0.59	F1 DURAT = 0.73/0.85, HSE = 0.91/0.94, LATEN = 0.36/0.39 F2 DISTB = 0.62/0.60, DAYDYS = 0.49/0.62	Second order model, No EFA	2; 2F-2	Compared with model reported in similar population, i.e., pregnant women	CFI & GFI > 0.90–1.0; RMSEA< 0.10 - < 0.05; χ^2^/df of 2 to 3 (lower is better); lower ECVI
Tomfohr et al. 2013 [36]	Mplus version 5.21	Maximum likelihood estimation	Reported but detail is not clear	Not reported, distinct model with age & gender as co-variates	F1 DURAT = 0.71/0.82, HSE = 0.70/0.72 F2 SLPQUAL = 0.77/0.76, LATEN = 0.64/0.63 F3 DISTB = 0.64/0.70, DAYDYS = 0.56/0.61	3, No EFA	3; 1F-1 3F-2	Not explained	CFI ≥ 0.90; SRMR ≤0.05; χ^2^ test of difference (*P* ≤ 0.01)
Zhong et al. 2015 [45]	SAS 9.4	Weighted least squares (WLS) estimation	Not reported	0.07, 0.36	F1 DURAT = 0.66, HSE = 0.52 F2 SLPQUAL = 0.47, LATEN = 0.46, DISTB = 0.45, DAYDYS = 0.64 F3 MEDS = 0.48 SLPQUAL = 0.22, LATEN = 0.26	3, same	5; 1F-1 2F-3 3F-1	Not explained, some of the documented models not used, no reasons given for selection and/or inclusion	CFI ≥ 0.90; SRMR< 0.08; RMSEA < 0.06
De la Vega et al. 2015 [59]	Not reported	maximum likelihood mean adjusted	Not reported	Not applicable	SLPQUAL = 0.421 LATEN = 0.620 DURAT = 0.656 HSE = 0.567 DISTB = 0.606 DAYDYS = 0.485	1, No EFA	2; 1F-1 2F-1	Compared with model reported in similar population, i.e., adolescents	S-Bχ^2^, CFI, RMSEA; cut-off for the indices not reported
Anandakumar et al. 2016 [67]	No CFA	–	–	–	–	–		–	–
Zheng et al. 2016 [51]	Not reported	Not reported	Not reported	0.34	F1 DURAT = 0.69 HSE = 0.65 MEDS = 0.15 F2 DISTB = 0.43 DAYDYS = 0.51 SLPQUAL = 0.721 LATEN = 0.63	2, No EFA	4; 1F-1 2F-2 3F-1	explained, some of the documented models not used, no reasons given for selection and/or inclusion	χ^2^, GFI, AGFI, RMR, RMSEA, CFI, NFI, NNFI, AIC, CAIC, SBC
Becker & Jesus 2017 [53]	SPSS 21 and AMOS-29	Not reported	–	–	F1 SLPQUAL = 0.59 LATEN = 0.76 F2 DURAT = 0.76 HSE = 0.69 F3 DISTB = 0.52 DAYDYS = 0.57	3, 2 different	6; 1F-2 2F-2 3F-2	Not explained, some of the documented models not used, no reasons given for selection and/or inclusion	non-significant χ2, RMSEA ≤0.08, CFi, GFI & AGFI > 0.97
Benhayon et al. 2013 [61]	No CFA	–	–	–	–	–	–	–	–
DeGutis et al. 2016 [62]	R	maximum likelihood estimation	Not reported	0.76, 0.75, 0.45	F1 HSE = 0.68 DURAT = 0.78 F2 LATEN = 0.70 SLPQUAL = 0.52 MEDS = 0.77 F3 DISTB = 0.56 DAYDYS = 0.78	No EFA	4; 1F-1 2F-2 3F-1	Not explained, some of the documented models not used, no reasons given for selection and/or inclusion	χ2/df < 3, SRMR & RMSEA≤0.06, CFI & TLI > 0 .95
Yunus et al. 2016 [48]	SPSS 20	weighted least squares method	Not reported	Not applicable	LATEN = 0.65 SLPQUAL = 0.65 DISTB = 0.49	1, No EFA	4; 1F-2 2F-1 3F-1	Not explained, some of the documented models not used, no reasons given for selection and/or inclusion	CFI, TLI, RMSEA, SRMR cut-off for the indices not reported
Qiu et al. 2016 [58]	SAS 9.4	weighted least squares (WLS) estimation	Error term correlation	0.68	F1 HSE = 0.48 DURAT = 0.45 LATEN = 0.44 SLPQUAL = 0.83 F2 DISTB = 0.62 DAYDYS = 0.49	2, same	6; 2F-6	None	CFI ≥ 0.90, SRMR≤0.08, RMSEA ≤0.06
Dudysova et al. 2017 [66]	Not reported	diagonally weighted least squares (DWLS) estimator	Not reported	0.80, 0.30, 0.16	F1 HSE = 0.68 DURAT = 0.88 F2 LATEN = 0.70 SLPQUAL = 0.79 MEDS = 0.89 F3 DISTB = 0.32 DAYDYS = − 0.29	No EFA	11; 1F-1 2F-6 3F-4	Not explained, some of the documented models not used, no reasons given for selection and/or inclusion	non-significant & lower, GFI > 0.90, CFI & TLI ≥0.95, RMSEA ≤0.05 (≤0.08 adequate fit), SRMR ≤0.08
Salahuddin et al. 2017 [16]	SPSS -16.0	maximum likelihood	Error term correlation	Not applicable	Not reported	1, 1–3	5; 1F-4 2F-1	All based on EFA	RMR & RMSEA ≤0.05, GFI, AGFI ≥0.90, Lesser ECVI, CFI ≥ 0.95, χ2/df ≤ 3
João et al. 2017 [57]	SPSS-21.0	No CFA	–	–	–	–	–	–	–
Chen et al. 2017 [63]	R 3.1.1 and its package lavaan	Not reported	Used modification indices but details not mentioned	Not reported	Unstandardized loadings Reported	None, No EFA	1; 3F-1	Not applicable	CFI & TLI > 0.90, RMSEA < 0.08
Khosravifar et al. 2015 [51]	Not reported	Not reported	Not reported	Not reported	Not reported	2	3; 1F-1 2F-1 3F-1	Based on EFA	Not reported
Fontes et al. 2017 [49]	STATA version, R, version 3.0.1	Not reported	Correlation between the PSQI components	Not applicable	HSE = 0.44 DURAT = 0.53 LATEN = 0.54 SLPQUAL = 0.88 MEDS = 0.22 DISTB = 0.42 DAYDYS = − 0.37	1, 2	2; 1F-1 2F-1	Based on EFA	non-significant χ2, χ2/df = 2–3, SRMR ≤0.08, RMSEA≤0.07, CFI & TLI ≥ 0.95
Guo et al. 2016 [60]	SPSS-22.0 with AMOS18.0	Not reported	Error term correlation	Not reported	HSE = 0.47 DURAT = 0.52 LATEN = 0.41 SLPQUAL = 0.83 DISTB = 0.35 DAYDYS = − 0.60	2, No EFA	6; 1F-2 2F-2 3F-2	Not explained, some of the documented models not used, no reasons given for selection and/or inclusion	χ2/df = 2–5, 0.05 < RMSEA < 0.08, CFI > 0.95, SRMR< 0.05
Morris et al. 2017 [65]	SPSS-22.0	No CFA	–	–	–	–	–	–	–
Passos et al. 2016 [52]	SPSS-20.0 with AMOS 23.0	Not reported	Error term correlation	0.17	Unstandardized loadings Reported	2–3, 2	3; 2F-2 3F-1	Based on EFA	SRMR≤0.08, CFI > 0.95, 0.5 < RMSEA> 0.8
Zhu et al. 2018 [64]	Stata 13.1	Maximum Likelihood Algorithm	Not reported	Not applicable	HSE = 0.81 DURAT = 0.75 LATEN = 0.61 SLPQUAL = 0.63 DISTB = 0.46 DAYDYS = − 0.43	1, No EFA	3; 1F-2 3F-1	Not explained, some of the documented models not used, no reasons given for selection and/or inclusion	non-significant χ2, RMSEA < 0.05, CFI > 0.95, lower BIC, SRMR< 0.06

## Results

### Sample description, sample size, and measures of the suitability of the data for factor analysis

The factor analysis of the PSQI has been reported on diverse samples including university/college students in Nigeria, India, Chili, Chinese, Ethiopia, Peru and Thailand, pregnant women, community dwelling adults and older adults in America, Australia, China, Ethiopia, Spain, Portugal, Iranian health professionals, adolescents and young adults [15–17, 23, 24, 26–28, 31, 34, 36, 39, 41, 44, 45, 52, 53, 57–60]. Moreover, it has been reported for patient population with breast cancer, Crohn’s disease, depression, diabetes, women with hot flashes, Taiwanese insomniacs, post-traumatic stress disorder, Trauma exposed veterans, temporo-mandibular disorder, traumatic brain injury, fibromyalgia, arthritis, chronic fatigue syndrome, psychiatric disorders and renal transplant patients [25, 27, 29, 30, 32, 33, 35, 37, 38, 40, 42, 43, 46, 49, 51, 61–65].

Few of the studies reviewed had large sample sizes (Table 1). The sample sizes of studies reporting the PSQI factor structures differed widely, ranging from 105 to 2840 (Table 1) [38, 44, 66]. Only 3 studies reported determinant scores and anti-image values (Table 1) [15–17]. We counted the number of studies reporting any of the following 5 indices: Kaiser-Meyer-Olkin (KMO) Measure of Sampling Adequacy, Bartlett’s sphericity test, determinant score, anti-image, and component correlations (Table 1). Eighteen studies reported both the KMO and Bartlett’s sphericity test [15–17, 25, 39, 43–45, 51–53, 57–59, 61, 64, 65, 67], reported inter-item correlations [16, 22, 24, 25, 27, 29, 30, 32–34, 36, 37, 41, 42, 45, 46, 50, 53, 57, 64–67], and 11 studies did not report any index [23, 26, 28, 31, 35, 38, 40, 49, 60, 62, 63].

### Exploratory factor analysis

Twenty articles did not report having carried out EFA [15, 23, 26, 29, 32–38, 42, 48, 50, 59, 60, 62–64, 66]. The types of EFA tests used were principal component analysis (PCA) in 18 studies [17, 22, 24, 25, 27, 28, 31, 39, 41, 43–45, 49, 51, 57, 58, 65, 67, 68], maximum likelihood estimation (MLE) in 6 [16, 17, 22, 24, 40, 41, 53] and unreported in 2 [30, 52] (Table 2). The types of rotation used for EFA were orthogonal varimax in 8 studies [28, 39, 41, 43, 44, 46, 49, 52, 65], oblique direct oblimin in 8 [16, 17, 22, 24, 25, 51, 53, 61], oblique promax in 5 [40, 45, 46, 58, 65], standardized geomin 1 [30], and unreported in 4 [27, 31, 57, 67]. Few of the studies justified the type of extraction or rotation used. Seven studies reported using a Cattell’s scree test to determine the number of factors to retain [16, 17, 44, 46, 58, 61, 65, 69]. Fifteen studies employed the Kaiser criterion of eigenvalue greater than one [16, 17, 25, 27, 28, 40, 43–46, 49, 51, 58, 61, 65].

There is increasing consensus among statisticians that, parallel analysis and Velicer’s minimum average partial (MAP) test, are better than to other procedures. This is because these methods usually give optimal solutions for the number of factors to retain [70]. However, only 4 studies used parallel analysis [16, 17, 49, 65, 71]. Fifteen studies used multiple criteria for factor retention [16, 17, 25, 27, 40, 43–46, 49, 51, 52, 58, 61, 65]. The number of factors retained after EFA varied across articles. Five studies reported a 1-dimensional PSQI structure [16, 17, 46, 52, 67], 17 reported a 2-dimensional structure [16, 17, 22, 24, 25, 27, 28, 30, 40, 43, 44, 49, 51–53, 58, 61], and 8 reported a 3-dimensional structure [16, 31, 39, 41, 44, 45, 52, 65]. Twenty studies reported the cumulative percent of variance extracted [16, 17, 22, 25, 27, 39, 40, 43, 45, 49, 51–53, 57, 58, 65, 67]. Only 4 studies reported the value of communality retention criteria of items for factor analysis [17, 25, 43, 59].

### Confirmatory factor analysis

As in the investigation of EFA, the overall finding for CFA was that a broad range of analytic techniques was used, all yielding a variety of inferred factor models. Eleven studies did not use CFA [25, 27, 28, 30, 31, 39, 43, 57, 61, 65, 67] (Table 3). Several different software programs were used for CFA: 6 studies used Mplus [26, 29, 34–36, 42], 12 used SPSS with Amos [15–17, 24, 33, 40, 48, 52, 53, 57, 60, 65], 4 used STATA [44, 46, 49, 64] and 2 each used LISREL [32, 37], and SAS [45 [58]], 1 each used FactoMineR [41], EQS [38], and SEM [23]. Five studies did not report which software program was used [22, 50, 51, 59, 66]. Thirteen studies did not report the method of extraction [23, 24, 29, 34, 40, 41, 49–53, 60, 63]. Nine studies used weighted least squares methods [26, 32, 35, 37, 45, 46, 48, 58, 66], 8 employed MLE [15, 16, 33, 36, 38, 44, 59, 62], 2 used MLE with bootstrapping for smoothing multivariate non-normality [17, 22], and 1 used robust MLE [42] (Table 3). Most of the studies did not use a modification index [17, 24, 26, 33–35, 37, 38, 40, 41, 44–46]. Twelve studies reported the result of the suggestions of the modification indices, 2 used path diagram change [23, 29], 6 used correlations between error terms [15, 16, 32, 42, 58, 60], 1 used path diagram change with Lagrange modifiers [22], and 2 did not provide details [36, 63] (Table 3). The reported correlation values between the factors of the final CFA models varied from 0.07 to 1.0. Some studies failed to report correlation values between the factors [26, 36] (Table 3).

Eight studies found that a 3-factor model best explained the data [29, 35–37, 41, 45, 52, 53], Thirteen reported a 2-factor model [22, 24, 26, 32, 34, 38, 40, 50–52, 58, 60], and 9 reported a 1-factor model [15–17, 42, 46, 48, 49, 59, 64]. One study reported both 2-factor and 3-factor models, but in separate sample populations [44] (Table 3). Two studies reported second-order models; i.e., 2 or 3 first-order latent factors loaded on a higher-order factor [23, 33] (Table 3). Seven studies found the same PSQI structure with both EFA and CFA [40–42, 44–46, 58], while 3 derived different models from EFA and CFA [17, 22, 24] (Table 3). The medicine component of the PSQI was removed from the final models in some studies [23, 36, 38], while sleep quality component of the PSQI was not reported in the final model by 2 studies [23, 40] (Table 3). Two studies reported finding a 2-factor model with just 5 PSQI components [16, 23], while 1 study reported a model with only three PSQI components [48] (Table 3). Three studies reported final models with cross-loads [29, 45, 63]. Two studies reported non-standardized factor loadings, while 2 studies did not report the factor loadings (Table 3) [26, 29, 46, 63]. The studies showed little variation in number, types, and limit values of the fit indices used.

## Discussion

### Sample description, sample size, and measures of the suitability of the data for factor analysis

The gradual development of a heterogeneous multiple factor structure of the PSQI has often been defended by the complexities of sleep problems among diverse samples. However, there is no consensus about this assertion that complexities of sleep problems in diverse samples must result in multiple factor structure [15]. Moreover, this speculative presumption conveniently ignores to explain why the measured variables, i.e. individual items of the PSQI and the PSQI component scores cannot account for this complexity.

The appropriate sample size for factor analysis is a frequently debated topic among statisticians. There are disparate guidelines [72–74]. There are also different opinions on such issues as sample to variable ratio (N:p ratio) criteria [72, 75], the factorability of the correlation matrix [76, 77], use of the KMO/Bartlett’s Test of Sphericity [76, 78], and use of the determinant of the matrix and anti-image or diagonal element of the anti-correlation matrix [72]. The suitable data for factor analysis and replicable factor extraction may require large samples and the satisfaction of a number of conditions as determined by such measures as the KMO, Bartlett’s test, the determinant of the matrix, the anti-image of the anti-correlation matrix, and inter-component correlations [79]. A non-zero determinant of the matrix indicates the absence of multi-collinearity, meaning that linear combinations of items can form factors [80, 81].

The non-reporting of these conditions by the majority (29 out of 45) of studies may create doubt about the applicability of the reported factor structures, even in the study populations. Three studies reported all but inter-component correlations) [15–17], while 7 reported KMO, Bartlett’s test and inter-component correlations (Table 1) [25, 39, 43–45, 53, 57, 64, 65, 67]. The conclusions about dimensionality of the PSQI by some of these studies are limited by non-reporting of CFA (Table 3) [25, 39, 43], or non-reporting of EFA [15]. However, reporting of multiple sample size suitability indices by these studies indicate suitability of their data for factor analysis [15, 17, 25, 39, 43–45].

### Exploratory factor analysis

The non-reporting of EFA results is fundamentally contrary to recommended norms for factor analysis, a deficiency that is particularly important considering the debate about the number and patterns of common factors for the PSQI [4, 15, 17, 82]. Although the choice of extraction types for performing EFA is much-debated, though some prefer the use of principal axes for initial solutions [72]. The choice of the extraction method (principal axis or principal factors) may depend on the underlying data and the assumptions [60]. Many studies failed to report the final extraction method used in the EFA (Table 2). Four studies reported using MLE for the final extraction [17, 22, 24, 41], but 3 of these did not report the normality and/or skewness of the distribution of data being analyzed [22, 24, 41]. The extracted factors’ applicability seems unclear because MLE entails multivariate normality [83]. Two studies reported using the principal factors method and principal component factor analysis, the authors might have meant principal axis method and PCA, respectively [43, 46]. Under these circumstances, it is unsurprising that most of the studies did not explain the types of extraction used, plus most of the studies did not explain the choice of rotation.

Factor rotation increases interpretability by optimizing a simple structure with a distinct cluster of interrelated variables loading on the least number of latent variables [80]. Oblique rotations are better suited to accounting for the inter-relationships in the clinical data. They can be used even when the factors are not significantly correlated [81]. However, the use of rotation methods in the PSQI factor analysis studies is inconsistent. Of the studies reporting rotation methods, similar numbers used orthogonal and oblique rotations (Table 2). Some of the studies using orthogonal rotation did report the correlation value of the extracted factors [28, 30, 41, 43, 44]. The reported factor correlations were in the range of 0.1–0.9 [39, 41, 44]. Therefore, the factor correlation values of the various PSQI models do not seem to support the choice of orthogonal rotation methods.

There are many criteria for determining the number of factors to be retained from EFA. These include the Cattell’s Scree test, Kaiser Criterion of Eigenvalue greater than one, the percentage of cumulative variance explained, and robust measures such as Horn’s Parallel analysis, the Broken-Stick (B-S) criterion, and the minimum average partial (MAP) test [72, 84]. These tests have many limitations, and more so for the first three tests mentioned earlier. Therefore, the consensus opinion is to employ multiple criteria [72, 84]. It is perhaps concerning that only approximately one-third of the PSQI factor analysis studies used multiple criteria, and none used multiple robust measures (Table 2) [84]. The B-S criterion and MAP test were not used by any of the studies exploring the PSQI’s factor structure. The communality accounts for the variance of the common factors. Factor analysis aims to explain variance through common factors. Therefore communalities less than 0.2 are removed [80]. However, communality criteria were frequently under-reported in the studies investigating the PSQI’s factor structure (Table 2). These inconsistencies and discrepancies might explain the variation in the number of factors retained after EFA (Table 2) [4, 15, 17].

### Confirmatory factor analysis

For finding prospective models and validation of the dimensionality of a questionnaire tool in discrete populations, it is recommended that factor analysis studies use both EFA and CFA [80]. More than 68% of the studies investigating the PSQI’s factor structure employed either EFA or CFA. Some of the PSQI models are based only on EFA [25, 27, 28, 31, 39, 43], while some are based only on CFA [15, 23, 26, 29, 32, 34–36, 38, 42], neither of which is the recommended practice for performing factor analysis [85].

Another issue is the influence of user software. The software packages used to perform CFA (LISREL, Mplus, SAS, STATA, Amos, and EQS) differ with regard to estimation; path diagrams; availability of standard errors for standardized estimates, factor covariance, and factor correlations; availability of modification indices; and ability to handle different types (i.e., continuous and categorical) of measured and latent variables [68]. However, the fact that studies investigating the PSQI’s factor structure used different software for CFA should not affect the results, as there are only slight differences in the statistics reported by the various programs, but the solutions are comparable [15]. LISREL, Mplus, SAS and STATA can handle the PSQI component scores, which are ordered as categorical variables, using diagonally weighted least squares estimation methods. Amos cannot accurately estimate models because it treats the PSQI component scores as measured variables. This is especially true if the PSQI component scores’ distributions are characterized by skewness and kurtosis [17, 68]. However, Amos allows model estimation using MLE with bootstrapping to smooth non-normality with standardized estimates of factor loading [86]. Non-reporting of distribution characteristics is a common problem with the PSQI factor analysis studies. Further, some studies using SPSS with Amos did not describe their extraction and bootstrapping methods [24, 40]. More than a quarter of the studies (i.e.13 out of 34 studies that used CFA) failed to report their extraction methods (Table 3). It is therefore difficult to reach a conclusion about the applicability of these studies’ results. Modification indices should be used discretely to avoid over-capitalization on sample specific variations. It may be better to validate the modification index incorporated models on unrelated samples [87]. Few studies reported using the modification index, and they did not explain the choice of the type of modification index [23, 29, 32, 36, 42].

Inter-factor correlation of 0.85 and above arises from multicollinearity and indicate poor discriminant validity [88]. The reported correlation coefficients between CFA model factors were as high as 0.89, 0.9, and 1.0 [33, 41]. This is technically undesirable because correlation coefficients greater than 0.9 suggest that the 2 correlated factors might not be practically distinct. Instead, the items loading on them might load on a common factor [17]. Jomeen and Martin [26] did not report inter-factor correlations in their final model. Moreover, they failed to report the factor loadings (Table 3). It is therefore difficult to reach a conclusion about their model’s parsimony.

Low loadings for some of the PSQI components’ scores (i.e., medicine component and sleep quality component) in some studies might reflect a reduced sensitivity of the questionnaire items measuring them [23, 36, 38, 40]. Tomfohr et al. [36] reported only the inter-component correlations as a sample size adequacy measure, did not use EFA, and did not provide details regarding the modification index. Among all the studies, Dudysova et al. [66] had the smallest sample size at 105. They did not report their EFA findings, nor did they provide information regarding suitability of the data for factor analysis, such as the KMO test, Bartlett’s test, determinant score, nor anti-image matrix. Similarly, Skouteris et al. [23] did not report their findings regarding EFA or sample size adequacy measures. They also did not report the CFA extraction method. Lequerica et al. [40] did not report any sample size measures or the CFA extraction method. The study used Amos without reporting normality conditions or bootstrapping.

Methodological discrepancies between these studies might have affected their results and the reliability of their findings. The model fit indices were streamlined with regard to number, types, and limit values (Table 3). Almost all the reviewed studies used multiclass model fits, which is consistent with generally accepted guidelines for factor analysis [89]. Gelaye et al. reported using 4 model fit indices in their study but mentioned the cut-off criteria of only 3. A model fit was presented for a 2-factor solution, though the EFA supported a 3-factor model [17, 44]. It is also concerning that in almost all the studies, the basic parsimony requirements for factor analysis were not upheld [15, 17]. It is worth noting that the recommended practice for factor analysis gives preference to parsimonious models over multidimensional models if differences are irreconcilable [47]. Therefore, the non-application of parsimony, together withother procedural discrepancies, has made it difficult to endorse the applicability of the various PSQI factor structures, even in similar samples.

### Practice points for future

- The studies investigating factor analysis of a questionnaire should employ both EFA and CFA.
- The reporting of details of sample suitability for factor analysis is preferable. This gives supporting evidence about distribution, levels of multicollinearity, singularity, and shared variance among measured variables.
- The details of EFA like extraction methods, rotation and factor retention should be reported along with their justification.
- The reporting of CFA like extraction methods and modification indices is preferable along with their justification.
- It is preferable to employ multiple goodness of fit indices from different categories.

### Limitations

This review has some limitations. We did not perform a meta-analysis, but the discrepancies made that almost impractical. The studies’ methodological qualities were not assessed, but such approaches have their own demerits [1]. We mostly reviewed articles published in the English language; with only 2 non-English articles included after their authors approved/provided a translation of the factor analysis sections [27]. Some authors did not respond to the queries regarding details of the factor analysis in their study. The authors of the other included articles were not contacted. Model fit indices were not discussed in detail because the studies were methodologically sound in this regard. Interested readers are referred Cheung and Rensvold [90].

## Conclusion

The results of this review do not permit an optimistic conclusion regarding the applicability of factor analysis studies on this widely used questionnaire. The generalizations from the majority are severely limited by issues including non-application of parsimony, non-use of EFA or non-reporting of relevant details, and non-use of CFA or non-reporting of relevant details. The generalizations from studies using small size may be difficult. Furthermore, under- or non-reporting of sample adequacy measures “and” non-reporting of relevant details make understanding the diversity of factor structures difficult to interpret. In summary, the factor analysis may not be replicable across different methodologies. The structured categorical data of the PSQI may be sensitive to the specific model (method of extraction) being applied.

Therefore, the applicability of the various PSQI factor structures even in related samples seems doubtful.
